# Housekeeping Mutualisms: Do More Symbionts Facilitate Host Performance?

**DOI:** 10.1371/journal.pone.0032079

**Published:** 2012-04-16

**Authors:** Adrian C. Stier, Michael A. Gil, C. Seabird McKeon, Sarah Lemer, Matthieu Leray, Suzanne C. Mills, Craig W. Osenberg

**Affiliations:** 1 Department of Biology, University of Florida, Gainesville, Florida, United States of America; 2 Laboratoire d’Excellence “CORAIL”, USR 3278 CNRS-EPHE, CBETM de l’Université de Perpignan, Perpignan, France; 3 Smithsonian Institution Marine Science Network, Smithsonian Marine Station, Ft. Pierce, Florida, United States of America; University of Arizona, United States of America

## Abstract

Mutualisms often involve one host supporting multiple symbionts, whose identity, density and intraguild interactions can influence the nature of the mutualism and performance of the host. However, the implications of multiple co-occurring symbionts on services to a host have rarely been quantified. In this study, we quantified effects of decapod symbionts on removal of sediment from their coral host. Our field survey showed that all common symbionts typically occur as pairs and never at greater abundances. Two species, the crab *Trapezia serenei* and the shrimp *Alpheus lottini*, were most common and co-occurred more often than expected by chance. We conducted a mesocosm experiment to test for effects of decapod identity and density on sediment removal. Alone, corals removed 10% of sediment, but removal increased to 30% and 48% with the presence of two and four symbionts, respectively. Per-capita effects of symbionts were independent of density and identity. Our results suggest that symbiont density is restricted by intraspecific competition. Thus, increased sediment removal from a coral host can only be achieved by increasing the number of species of symbionts on that coral, even though these species are functionally equivalent. Symbiont diversity plays a key role, not through added functionality but by overcoming density limitation likely imposed by intraspecific mating systems.

## Introduction

Mutualisms are widespread across taxa, contributing to the structure and function of ecosystems [1], [2], [3], especially when the mutualisms involve foundation species such as trees or stony corals. Historically, mutualisms have been studied through pairwise interactions, for example, between a single symbiont and a single host. However, many hosts are inhabited by multiple species of potentially interacting symbionts [4], [5] necessitating the need to understand how multiple co-occurring symbiont species influence patterns of association within the symbiont guild, and how effects of symbiont species combine to influence the host [6], [7], [8], [9].

Co-occupation of hosts by multiple symbionts creates opportunities for emergent effects on the host that potentially go beyond effects anticipated based upon pairwise interactions (i.e. higher-order interactions, [10]). For example, experimental studies of predator-prey interactions have evaluated how the combined effects of two or more predator species compare to those predicted based upon pairwise effects of a single predator species [11]. These “multiple predator effect” studies evaluate if effects are independent (e.g., the predator species do not interact), synergistic (i.e., risk to prey is greater than expected), or inhibitory (e.g., predators incur intraguild predation or interference and thus risk to prey is reduced) [12]. A comparable framework may prove valuable for understanding symbiont effects [9], [13], and thus may help explain patterns of symbiont association and host responses to shifts in symbiont density and diversity. For example, antagonism between pollinator species can reduce the pollination services provided to plants by subordinate pollinators [7].

Insights from such experiments can evaluate putative links between biodiversity of the symbionts and ecosystem function mediated through host responses, as has been suggested in studies of myrmecophytic ants that defend plants [14], [15], snail grazers that remove epiphytes on algae [16], bacterial endosymbionts that convey antibiotic resistance to corals [17], and microbes that supply nitrogen to plant roots [18], [19].

Tropical coral reef ecosystems, with their exceptional biodiversity, are ideal models for studying mutualisms. Many reef corals house symbionts within tissues (i.e., endosymbionts, such as zooxanthellae) and within their branches (i.e., exosymbionts, such as some fishes and invertebrates). These symbionts depend critically upon the coral for their existence. For example, in the Indo-Pacific, some species of trapeziid crabs and alpheid shrimps live exclusively within the branches of pocilloporid corals [20]. Furthermore, work has shown that these decapod symbionts increase their coral host’s survival and growth by defending them from corallivorous sea stars and gastropods [13], [21], [22], removing mucus nets produced by vermetid gastropods [23], and removing sediment deposited on the coral tissue [24]. Only one study has examined how >1 species of exosymbiont combine to influence coral hosts: McKeon et al. [13] suggested that effects of two coral symbionts (*Trapezia serenei* and *Alpheus lottini*) combined synergistically to protect the host coral from seastar predators.

Sediment removal by coral symbionts may be particularly critical to the resilience of reef ecosystems. Natural disturbances (e.g. cyclones and storms) and coastal urbanization cause terrestrial runoff and re-suspension of bottom sediments, which increase sediment deposition onto corals [25]. Sedimentation decreases growth and increases mortality of corals [26], [27]. Although corals can remove some sediments from their surface through mucus sloughing, cilia movement, or polyp extension [28], [29], [30], symbionts potentially play a critical role in protecting corals from the deleterious effects of sedimentation by removing additional sediment [24].

Here, we document patterns of association between symbiont species occurring in *Pocillopora* coral colonies. We then use an experimental design and analytical approach borrowed from the predator-prey literature [31] to quantify the separate and combined effects of the two most common and positively associated symbiont species on sediment removal from corals.

## Materials and Methods

### Field Survey

Our study was conducted on Moorea, French Polynesia, a high island surrounded by a barrier reef. Over three consecutive days (July 19–21, 2010), we conducted a field survey on the western shore of Moorea near the reef crest (17°32′20′′S, 149°54′34′′W and 17°34′05′′S, 149°52′50′′W). We collected colonies of *Pocillopora cf. verrucosa* (hereafter *Pocillopora*) ranging from 45–160 cm maximum diameter from 1–2 m depth by enveloping each colony in a plastic bag to prevent symbiont loss, and removing each colony from the substrate with a chisel. Corals were immediately transported in coolers to the laboratory, where we measured each coral (maximum diameter, perpendicular diameter, maximum height and circumference) and removed all coral-dwelling fishes and decapods. We identified all decapods larger than ∼4 mm in carapace length and retained corals and decapods for later use in the experiments (see below). We focused on trapeziid crabs and alpheid snapping shrimps because they were common, conspicuous, readily identifiable, and are known to play an important functional role in the growth and survival of *Pocillopora* [21].

### Sediment Removal Experiment

We conducted an experiment in four large flow-through outdoor seawater tanks (2670 l; 3 m diameter), with the experiment repeated across four consecutive nights (each tank and night comprised a block). For each block, we selected five corals for similarity in size, branching morphology, and color. All corals came from the field survey, had originally contained symbionts, and had been collected within 48 h prior to their use in the experiment. We used two species of symbionts: *Alpheus cf. lottini ‘stripes’* (hereafter *Alpheus lottini*: see Table S1 *)* and *Trapezia serenei*. Each tank contained a single replicate of each of five treatments, which were assigned at random within each block: Control (0 symbionts); AA (2 *Alpheus lottini*); TT (2 *Trapezia serenei*); AT (1 *A. lottini* and 1 *T. serenei*); AATT (2 *A. lottini* and 2 *T. serenei*). Thus, the experiment combined an additive and substitutive design [31]: the additive design controlled intraspecific density, and the substitutive design controlled total symbiont density.

Intraspecific pairs of decapods collected from the field survey were maintained in flow-through containers for up to 48 h prior to the start of the experiment. For the AA, TT, and AATT treatments, intraspecfic pairs were collected from the same coral. We retained intraspecific pairs for these treatments because previous studies have shown that *Trapezia* conspecifics form reproductive pairs and that these pairs can inhibit recruitment by conspecifics [32]. For the AATT treatment, the *Trapezia* and *Alpheus* came from different corals. For the AT treatment, pairs of each species were separated for use in different corals. Otherwise, symbionts were assigned to treatments randomly.

Sediments were collected from beaches on the north shore of Moorea, passed through soil sieves to isolate the 2–2.5 mm fraction, dried at 70°C overnight, and divided into 50 g aliquots. Using cinder blocks, corals were suspended atop a rigid plastic grid (mesh size  =  1 cm) above sediment collection bins; 60 cm separated adjacent units within a tank (Fig. S1). Corals and their symbionts were added to the experimental tanks approximately 8 h and 1 h, respectively, prior to sediment addition. At dusk, sediment trials were initiated by adding 50 g of sediment to each coral colony simultaneously within blocks. Sediment that did not settle onto the coral colony was collected in bins and discarded, and the collection bins were immediately returned to collect the sediment removed during the night, the period over which the decapods are most active [33]. At dawn (∼13 hours after trial initiation) sediment bins were recollected. Sediment retained on each colony was also collected. All collected sediments were dried and weighed. The proportion of sediment that remained on the coral was determined as the dry mass of the remaining sediment divided by the sum of the dry masses of the remaining sediment and that collected at dawn in the bin. All necessary permits were obtained from the Délégation à la Recherche de la Polynésie française for the described field studies.

### Statistical Methods

#### Natural Variation in Richness, Co-occurrence, and Intraspecific Abundance

To determine whether observed patterns of species richness, co-occurrence, and abundance differed from patterns expected by chance, we generated null distributions using the five most abundant species. For species richness and co-occurrence, we fixed the number of corals (out of 133) that were actually occupied by each of the species, but randomly assigned each species to the 133 corals. We repeated the process for a total of 10,000 iterations. To assess patterns of abundance within each species (e.g., to assess if intraspecific pairs occurred more often than expected by chance), we fixed the total number of individuals of each species (at the observed number), but randomly allocated those individuals to the 133 corals. This was repeated 10,000 times for each species. We then determined the upper and lower 2.5% quantiles for each occurrence combination and compared our observed frequencies to those null intervals.

#### Sediment removal experiment

Effects of symbionts on sediment retention were quantified using a linear mixed effects model (fixed effect: symbiont treatment; random effect: experimental block) with four orthogonal contrasts: 1) symbiont effect (symbionts absent (Control) vs. present (AA, TT, AT, AATT)), 2) density effect (two symbionts (AA, TT, AT) vs. four symbionts (AATT)), 3) complementarity effect (monospecific pairs (AA and TT) vs. multi-species pairs (AT), and 4) identity effect (*Alpheus* (AA) vs. *Trapezia* (TT)). Proportion of sediments remaining on the coral was arcsine-square root transformed prior to analysis to increase normality and reduce heteroschedasticity.

We developed an analytical approach similar to that used in “Multiple Predator Effect” (MPE) studies [11] to estimate the interactions between symbionts on sediment removal. Three separate null models were used to estimate the expected level of sediment retention in the presence of both species of symbionts (i.e., treatments AT and AATT), assuming that the symbionts had independent effects on sediment removal. For example, assuming that effects of symbionts combined independently, then the expected retention (i.e., “survival”) of sediment in the AT treatment (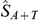) can be predicted from the single species results:
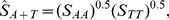
(1)where *S_i_* is the average proportion of sediment retained on the coral in the *i* ^th^ experimental treatment, and the carat indicates an expected proportion.

The expected retention of sediment in the AATT treatment can be generated in two ways. Based on results from the monospecific treatments (AA and TT), the null expectation is:
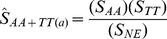
(2)Using results from the multi-species treatment (AT) yields:
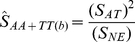
(3)To generate confidence intervals, we randomly sampled the observed data with replacement and calculated the null expectation based upon the two “observed” samples using Equation 2 (or 3). We repeated this 10,000 times and determined the 95% quantiles, which were then compared to the observed result (i.e., 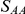 or 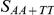). Significant deviations of the observed values from the distribution of expected values would suggest either synergy (sediment survival was worse than expected) or interference (sediment survival was greater than expected) between the symbiont species.

## Results

### Field Survey

**Figure 1:**
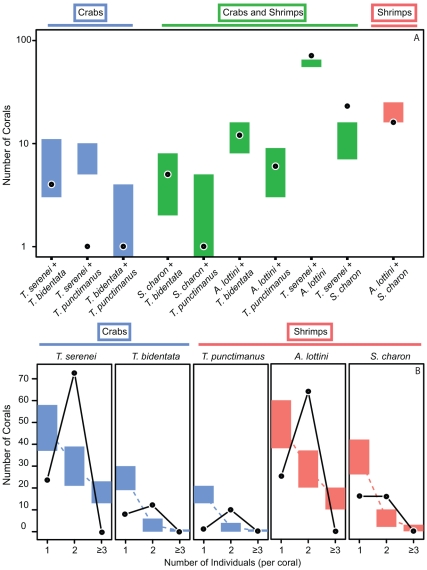
Interspecific co-occurrence (a) and intraspecific density (b) of five focal symbiont species from 133 surveyed corals. Pairwise co-occurrence of five focal symbiont species are shown within Trapezidae (i.e. Crabs – blue), within Alpheidae (i.e. Shrimps – pink), between the two families (Crabs and Shrimps – green). Black circles and solid line represent the observed data. Colored rectangles and dashed lines represent the 95% quantiles from 10,000 randomly simulated communities. Observed values are significantly different from the randomly simulated communities when the black circle falls outside the 95% quantiless. Species tend to avoid to avoid confamilials (with 2 of 4 comparisons demonstrating avoidance), while the crab, *T. serenei* is positively associated with both shrimp species. At the intraspecific level, pairs are more common and singlets and triplets are more rare than expected by chance. See Appendices B and E for raw data.

A total of 11 species of trapeziid and alpheid decapod symbionts >4 mm carapace length were collected from the 133 surveyed corals (see Table S1 for a species list). Five species were sufficiently abundant to analyze further (each occurring in >10 corals). 129 (97%) of the 133 surveyed corals contained at least one of these five focal species (Fig. S2). A majority of corals contained two (of the five) species of symbiont, which was more common than expected by chance (Fig. 1a, Table S2). The high frequency of corals occupied by just two species of symbionts was driven primarily by *Trapezia serenei*, which was positively associated with both species of alpheid shrimps (*A. lottini* and *Synalpheus charon*) (Fig. 1a). This positive association between crab and shrimp species was not observed for the other *Trapezia* species. Furthermore, species of the same family tended to avoid one another. The two shrimp (*A. lottini* and *S. charon*) and two of the crabs (*T. serenei* and *T. punctimanus*) co-occurred less often than expected by chance. The two other crab-crab associations also suggested avoidance, although the patterns did not differ significantly from those expected by chance (Fig. 1a).

Similarly, intraspecific pairs of symbionts occurred at high frequency. Of the 424 individual symbionts collected (of the five focal species), 41% occurred as intraspecific pairs (Table S2). Intraspecific density consistently deviated from the random expectation, with pairs of individuals occurring most frequently for all five abundant taxa (Fig. 1b, Table S3). We never observed more than two individuals of a species in any coral colony.

In summary, the two most abundance species, *T. serenei* and *A. lottini*, each occur in intraspecific pairs and co-occur more often than expected by chance. We therefore hypothesized that the co-occurrence of these two species might lead to a synergism in the ecological services they provide to the shared *Pocillopora* host.

### Sediment Removal Experiment

**Figure 2:**
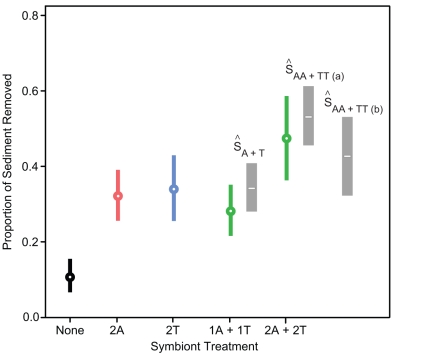
Independent effects of symbionts (*Alpheus*: A and *Trapezia*: T) on the proportion of sediment removed in the experiment. Figure shows backtransformed mean ± 95% CI, n  =  13. Grey vertical bars are give the 2.5% and 97.5 quantiles for the distribution of effects of symbionts assuming independence and calculated from equations 1, 2, and 3. The white bar in center of each grey bar is the mean from these simulations. Observed values fall within the null distributions for both the additive and substitutive designs suggesting that the effects of symbionts are independent (i.e. the data provide no evidence for antagonism or synergy between symbiont species).

We statistically modeled sediment retention (“survival”) on the coral using a framework adapted from studies of prey survival from the multiple-predator-effects literature. However, hereafter we present the data in the form of sediment removal (i.e. 1 – (proportion sediment retained)) because removal more clearly emphasizes the beneficial effect on the coral host. In the absence of symbionts, the coral in combination with physical disturbance reduced sediment loads by ∼10% (i.e. ∼90% of the sediment remained on the coral). This removal rate was small relative to that of the corals containing symbionts, which increased the absolute amount of sediment removal to ∼35%. This positive effect of symbionts on sediment removal increased with a doubling in symbiont density (i.e., from ∼32% to 48% (*t_1,36_*  =  –3.87, *p*  =  0.001). There was, however, no evidence for complementarity between symbionts (*t_1,36_*  =  –0.88, *p*  =  0.385) or differences between symbiont species (*t_1,36_*  =  –0.66, *p*  =  0.601): Fig. 2.

The two combined-species treatments (i.e., AT and AATT) did not differ significantly from that expected if their effects combined independently (Fig. 2). Thus there was no evidence for synergy or interference between these symbionts.

## Discussion

Symbionts substantially enhanced sediment removal from *Pocillopora*. The mechanism(s) by which symbionts remove sediment remains unclear, however. Three possibilities exist: 1) active removal where symbionts pick off grains of sediment, 2) passive removal, where sediment is removed during movement of symbionts, or 3) symbionts facilitate the coral’s own sediment removal ability (e.g. by stimulating mucus sloughing, cilia movement, or polyp extension).

Previous studies have obtained mixed results about interspecific variation in symbiont effects on hosts. Some, like our study, found that two symbiont species have similar effects on their host, while other studies have found demonstrable differences among species [16], [34]. For example, in another crab-coral system, McKeon [35] conducted single species trials with three species of *Trapezia* and found that one species removed significantly more sediment than the other two. Despite this work on pairwise interactions, very little work has determined if pairwise results can be extrapolated to multiple symbiont effects, although these situations are common and have the potential to modify the dynamics of foundation species. If interactions within a symbiont guild lead to synergy (or antagonism) of effects, host-symbiont dynamics and co-evolution may be different than expected from results of pairwise experiments. The few existing multi-symbiont studies have shown both synergism [13], [16], [36] and antagonism [17], [37]. In contrast, we found that effects combined independently. In a previous study in the same system as ours, McKeon et al. [13] found that effects of *T. serenei* and *A. lottini* combined synergistically to deter coral predators. This diversity of results suggests there is no general pattern across multi-symbiont systems or even within the same system.

When different species perform similar tasks in a system (e.g., sediment removal), this functional redundancy can buffer ecosystem services to the loss of one or more species [38]. However, this buffering often requires an increase in the density of remaining species [39]. In our system, however, we never observed >2 individuals of the same species per coral (Fig. 1b). This likely reflects the formation of reproductive pairs and their inhibition of recruitment by other individuals (e.g., as has been shown in two *Trapezia* conspecifics: *T. intermedia* and *T. digitalis*) [32]. Thus, the buffering effect that protects ecosystem services can be lost in systems such as ours [32], [40]: because of intraspecific competition, the only way to effectively increase symbiont density is to increase the number of species inhabiting a coral. Indeed, in our survey, diversity and abundance were positively correlated (Fig. S3). Therefore, symbiont diversity may remain important to the host despite functional redundancy among symbionts.

Larger *Pocillopora* corals generally support more species [41], [42] and they likely also support more pairs within a species [42]. Thus, the constraint we have noted likely is reduced in larger colonies. Increases in coral host performance with higher symbiont diversities may lead to positive feedback, with an increase in coral size supporting an increase in symbiont diversity (and density). This also should expand the capacity for compensation by other species if one species is lost. Future work on this and other multi-symbiont systems will therefore require both the study of pairwise interactions (to quantify species effects) and multiple species effects (to evaluate how these effects combine), as well as attention to the patterns of host occupancy and symbiont-host dynamics (to assess how ecosystem services may be altered by shifts in the symbiont community).

## Supporting Information

Figure S1 **Panel A shows a** ***Pocillopora*** **coral being transported in a plastic container.** After the coral was transferred to the red plastic grid, the container was placed underneath the grid to capture sediment removed by the coral and exosymbionts. Panel B shows a close up of a replicate coral with both *Trapezia serenei* (top) and *Alpheus lottini* (bottom).
(TIF)

Figure S2 **Frequency of species richness on 133 surveyed corals for top five focal exosymbionts (** ***Trapezia serenei*** **,** ***Alpheus lottini*** **,** ***Synalpheus charon*** **,** ***T. bidentata*** **,** ***T. punctimanus*** **).** Black circles and solid line represent the observed data. Purple rectangle and dashed line represent the 95% quantiles of richness from 10,000 randomly simulated communities. Pairs of species occur more frequently than expected by chance.
(EPS)

Figure S3 **The diversity of crustacean communities increases with total abundance.** Here we show the abundance-diversity relationship extracted from papers on communities inhabiting *Pocillopora damicornis* [43] (red - a), *Stylophora pistillata* [44] (green – b), and our study in *Pocillopora cf. verrucosa* (blue - c). Each point gives the species richness and abundance for a single coral colony. Because the two previous studies describing this relationship included juvenile crustaceans, we have included juveniles in our data set as well (excluding juveniles from our data, still leads to a positive correlation; *p* < 0.001, n  =  133, *r^2^*  =  0.77). Note the log_10_ scale on the x and y-axis.
(EPS)

Table S1 **Species list, number of corals occupied, and total abundance of exosymbionts from Alpheidae and Trapeziidae from surveys of 133 corals.**
(DOC)

Table S2 **Occurrence and co-occurrence patterns of five focal species (** ***Trapezia serenei*** **,** ***Alpheus lottini*** **,** ***Synalpheus charon*** **,** ***T. bidentata*** **,** ***T. punctimanus*** **) on 133 surveyed reefs.** Simulation quantiles represent 95% confidence interval from 10,000 randomly generated communities.
(DOC)

Table S3 **Abundance of individuals within a given species across all 133 corals for top five focal species of exosymbiont.** Simulation quantiles represent 95% confidence interval from 10,000 randomly generated communities.
(DOC)
