# Supplementary figures and images for: Housekeeping Mutualisms: Do More Symbionts Facilitate Host Performance?
Source: PLoS One. 2012 Apr 16;7(4):e32079. doi: 10.1371/journal.pone.0032079 (PMC3327697; doi:10.1371/journal.pone.0032079)

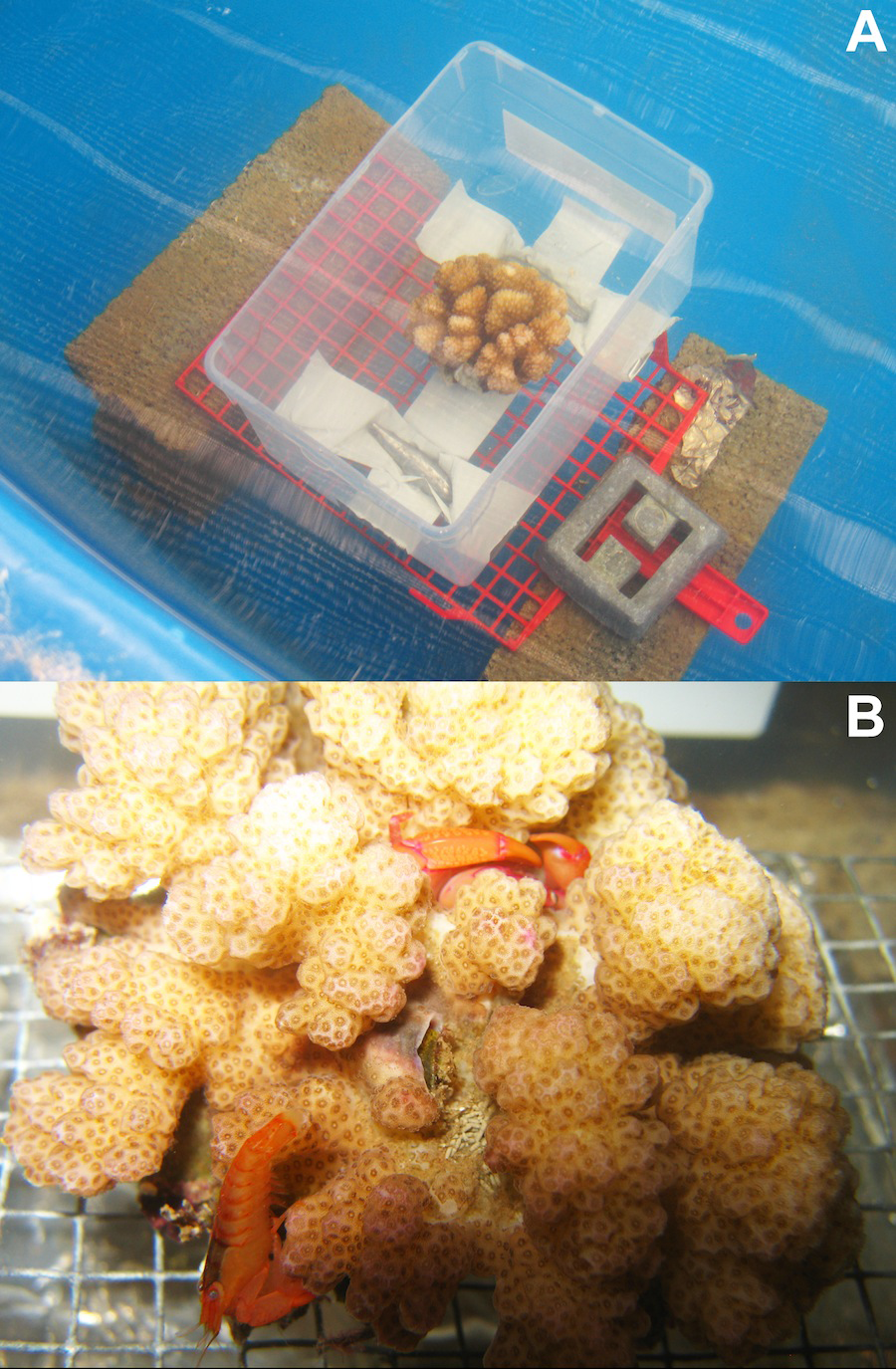

Supplement: Figure S1 — Panel A shows a Pocillopora coral being transported in a plastic container. After the coral was transferred to the red plastic grid, the container was placed underneath the grid to capture sediment removed by the coral and exosymbionts. Panel B shows a close up of a replicate coral with both Trapezia serenei (top) and Alpheus lottini (bottom). (TIF) [file pone.0032079.s001.tif]
