# Supplementary material for: Housekeeping Mutualisms: Do More Symbionts Facilitate Host Performance?
Source: PLoS One. 2012 Apr 16;7(4):e32079. doi: 10.1371/journal.pone.0032079 (PMC3327697; doi:10.1371/journal.pone.0032079)
Supplement: Table S1 — Species list, number of corals occupied, and total abundance of exosymbionts from Alpheidae and Trapeziidae from surveys of 133 corals. (DOC) [file pone.0032079.s004.doc]

Table S1

| Decapod Species | Number of Corals | Total Abundance |
| --- | --- | --- |
| *Trapezia serenei* | 97 | 170 |
| *Alpheus c.f. lottini* ‘stripes’^1^ | 87 | 149 |
| *Alpheus c.f. lottini* ‘spots’^1^ | 2 | 4 |
| *Trapezia bidentata* | 20 | 32 |
| *Synalpheus charon* | 32 | 48 |
| *Trapezia punctimanus* | 11 | 21 |
| *Trapezia speciosa* | 3 | 5 |
| *Trapezia bella* | 3 | 4 |
| *Trapezia tigrina* | 1 | 2 |
| *Trapezia lutea* | 1 | 1 |
| *Trapezia areolata* | 1 | 1 |

^1^The taxonomic status of *Alpheus lottini* is currently in flux. Previous genetic analysis suggest *A. lottini* is a species complex [1], with at least two species in Moorea (A. Anker and C.S. M^c^Keon, personal communication). In Moorea two morphotypes of *Alpheus cf. lottini* exist with either stripes or spots on their carapace. While taxonomic designation of these two species has yet to be completed, throughout this manuscript we distinguish *A. lottini* 'stripes' from *A. lottini* 'spots'. Notably, *A. lottini* 'stripes' made up ~98% of the individuals sampled. In all other portions of this paper, including the appendices, *Alpheus* *lottini* refers only to ‘stripes’.

1. Williams ST, Jara J, Gomez E, Knowlton N (2002) The Marine Indo-West Pacific Break: Contrasting the Resolving Power of Mitochondrial and Nuclear Genes. Integrative and Comparative Biology 42: 941-952.
