# Supplementary material for: Housekeeping Mutualisms: Do More Symbionts Facilitate Host Performance?
Source: PLoS One. 2012 Apr 16;7(4):e32079. doi: 10.1371/journal.pone.0032079 (PMC3327697; doi:10.1371/journal.pone.0032079)
Supplement: Table S2 — Occurrence and co-occurrence patterns of five focal species ( Trapezia serenei , Alpheus lottini , Synalpheus charon , T. bidentata , T. punctimanus ) on 133 surveyed reefs. Simulation quantiles represent 95% confidence interval from 10,000 randomly generated communities. (DOC) [file pone.0032079.s005.doc]

**Table S4**

|  | *Species* | | | | |  | Simulation Quantiles | |
| --- | --- | --- | --- | --- | --- | --- | --- | --- |
| *Grouping* | *T. serenei* | *A. lottini* | *S. charon* | *T. bidentata* | *T. punctimanus* | Observed No. of Corals | Lower 2.5% | Upper 2.5% |
| Empty |  |  |  |  |  | 4 | 3 | 12 |
| Singlets |  |  |  |  |  |  |  |  |
|  | X |  |  |  |  | 14 | 14 | 26 |
|  |  | X |  |  |  | 1 | 9 | 19 |
|  |  |  | X |  |  | 3 | 0 | 5 |
|  |  |  |  | X |  | 6 | 0 | 4 |
|  |  |  |  |  | X | 3 | 0 | 2 |
| Pairs |  |  |  |  |  |  |  |  |
|  | X | X |  |  |  | 57 | 31 | 44 |
|  | X |  | X |  |  | 10 | 3 | 11 |
|  | X |  |  | X |  | 0 | 1 | 7 |
|  | X |  |  |  | X | 1 | 0 | 4 |
|  |  | X | X |  |  | 2 | 1 | 8 |
|  |  | X |  | X |  | 7 | 0 | 6 |
|  |  | X |  |  | X | 6 | 0 | 4 |
|  |  |  | X | X |  | 1 | 0 | 2 |
|  |  |  | X |  | X | 1 | 0 | 1 |
|  |  |  |  | X | X | 0 | 0 | 1 |
| Triplets |  |  |  |  |  |  |  |  |
|  | X | X | X |  |  | 11 | 7 | 17 |
|  | X | X |  | X |  | 2 | 3 | 11 |
|  | X | X |  |  | X | 0 | 1 | 6 |
|  | X |  | X | X |  | 1 | 0 | 3 |
|  | X |  |  | X | X | 0 | 0 | 2 |
|  | X |  |  | X | X | 0 | 0 | 2 |
|  |  | X | X | X |  | 2 | 0 | 3 |
|  |  | X | X |  | X | 0 | 0 | 2 |
|  |  | X |  | X | X | 0 | 0 | 1 |
|  |  |  | X | X | X | 0 | 0 | 1 |
| Quadruplets |  |  |  |  |  |  |  |  |
|  | X | X | X | X |  | 1 | 0 | 5 |
|  | X | X | X |  | X | 0 | 0 | 3 |
|  | X | X |  | X | X | 0 | 0 | 2 |
|  | X |  | X | X | X | 0 | 0 | 1 |
|  |  | X | X | X | X | 0 | 0 | 1 |
| Quintuplet |  |  |  |  |  |  |  |  |
|  | X | X | X | X | X | 0 | 0 | 1 |
