# Supplementary material for: Housekeeping Mutualisms: Do More Symbionts Facilitate Host Performance?
Source: PLoS One. 2012 Apr 16;7(4):e32079. doi: 10.1371/journal.pone.0032079 (PMC3327697; doi:10.1371/journal.pone.0032079)
Supplement: Table S3 — Abundance of individuals within a given species across all 133 corals for top five focal species of exosymbiont. Simulation quantiles represent 95% confidence interval from 10,000 randomly generated communities. (DOC) [file pone.0032079.s006.doc]

**Table S5**

|  |  |  | Simulation Quantiles | |
| --- | --- | --- | --- | --- |
| Species | No. Symbionts | No. Corals | Lower 2.5% | Upper 2.5% |
| *T. serenei* | 0 | 36 | 30 | 44 |
|  | 1 | 24 | 37 | 58 |
|  | 2 | 73 | 21 | 39 |
|  | ≥3 | 0 | 13 | 23 |
|  |  |  |  |  |
| *T. bidentata* | 0 | 113 | 102 | 108 |
|  | 1 | 8 | 19 | 30 |
|  | 2 | 12 | 0 | 6 |
|  | ≥3 | 0 | 0 | 1 |
|  |  |  |  |  |
| *T. punctimanus* | 0 | 122 | 112 | 116 |
|  | 1 | 1 | 13 | 21 |
|  | 2 | 10 | 0 | 4 |
|  | ≥3 | 0 | 0 | 1 |
|  |  |  |  |  |
| *A. lottini* | 0 | 44 | 35 | 49 |
|  | 1 | 25 | 38 | 60 |
|  | 2 | 62 | 20 | 39 |
|  | ≥3 | 0 | 10 | 20 |
|  |  |  |  |  |
| *S. charon* | 0 | 101 | 88 | 97 |
|  | 1 | 16 | 26 | 42 |
|  | 2 | 16 | 2 | 10 |
|  | ≥3 | 0 | 0 | 3 |
